# Exposure to a Mixture of Polychlorinated Biphenyls and Polychlorinated Dibenzofurans Resulted in a Prolonged Time to Pregnancy in Women

**DOI:** 10.1289/ehp.10715

**Published:** 2008-01-24

**Authors:** Chiu-Yueh Yang, Ying-Jan Wang, Pau-Chung Chen, Shaw-Jenq Tsai, Yueliang Leon Guo

**Affiliations:** 1 The Institute of Basic Medical Sciences, National Cheng Kung University Medical College, Tainan, Taiwan; 2 Department of Health Business Administration, Hung-Kuang University, Taichung, Taiwan; 3 Department of Environmental and Occupational Health, National Cheng Kung University Medical College, Tainan, Taiwan; 4 Department of Occupational Medicine and Industrial Hygiene, National Taiwan University, Taipei, Taiwan; 5 Department of Physiology, National Cheng Kung University Medical College, Tainan, Taiwan; 6 Department of Environmental and Occupational Medicine, National Taiwan University (NTU) and NTU Hospital, Taipei, Taiwan

**Keywords:** fertility, infertility, PCBs, PCDFs, polychlorinated biphenyls, polychlorinated dibenzodioxins, polychlorinated dibenzofurans, pregnancy, reproduction, time to pregnancy

## Abstract

**Background:**

Polychlorinated biphenyls (PCBs), dibenzofurans (PCDFs), and dibenzodioxins (PCDDs) may affect the female reproductive system in animals and humans. In 1978–1979, a mass poisoning occurred in central Taiwan due to PCB/PCDF-contaminated cooking oil; this incident was called Yucheng (“oil disease” in Chinese).

**Objective:**

The purpose of our study was to determine whether PCB/PCDF exposure affected fertility in exposed women.

**Methods:**

After the event, we followed the exposed individuals and a reference group who were sex-, age-, and community-matched. In 2003, we obtained fertility histories from Yucheng and reference women by telephone interview. We used Kaplan–Meier survival curves and multivariable Cox regression to compare time to pregnancy (TTP) between Yucheng and reference women, and we performed multiple logistic regression to determine whether PCB/PCDF exposure caused infertility.

**Results:**

In total, 412 women responded, with a median TTP of 4 months in Yucheng women and 3 months in reference women (*p* = 0.019). After adjusting for confounders by Cox regression, we found a fecundability ratio of 0.90 [95% confidence interval (CI), 0.80–1.00] for Yucheng women. Among the 408 women who had noncontraceptive sexual activity for > 12 months, 19.7% of Yucheng women and 9.7% of reference women did not become pregnant (i.e., they were infertile). After we adjusted for confounders by logistic regression, the infertility odds ratio was 2.34 (95% CI, 1.23–4.59) for Yucheng women compared with the reference group.

**Conclusions:**

We found prolonged TTP and reduced fertility among women previously exposed to PCBs/PCDFs. Because of the limited sample size and the relatively small decrease in the fertility rate, these effects require cautious interpretation and further investigation for confirmation.

Polychlorinated biphenyls (PCBs) have been extensively produced as technical mixtures and have been used as dielectric fluids in transformers and capacitors, organic diluents, plasticizers, adhesives, and flame retardants, as well as in several other industrial applications. Because of extensive use for several decades in the twentieth century, PCBs have become ubiquitous environmental contaminants. Furthermore, they are persistent environmental pollutants that bioaccumulate in the food chain and are difficult to metabolize in wildlife and humans. Therefore, PCBs have been detectable in humans even though PCB production and use has been banned or restricted in many countries since the late 1970s. Polychlorinated dibenzofurans (PCDFs) and polychlorinated dibenzodioxins (PCDDs) are by-products of human activities, primarily industry, agriculture, and incineration. However, because of their hormone-like activities and binding with the aryl hydrocarbon receptor (AhR) in humans and animals, these toxicants may cause significant health effects in spite of rather low exposure.

In animal studies, some noncoplanar PCB congeners and their hydroxylated metabolites were found to be weakly estrogenic (Arcaro et al. 1999; Gierthy et al. 1997). Conversely, dioxin-like chemicals, dibenzofurans, and coplanar PCBs were shown to be anti-estrogenic (Hombach-Klonisch et al. 2006). Therefore, the female reproductive system could be affected by exposure to these chemicals. PCBs decreased female conception rates and increased stillbirth in animals (Barsotti et al. 1976; Bleavins et al. 1980; Reijnders 1986; Wiemeyer et al. 1984). Only a few human studies have investigated the effects of exposure to PCBs or other persistent organochlorine compounds on female fertility, and the findings were inconsistent (Axmon et al. 2000; Cole et al. 2006; Courval et al. 1999; Gerhard et al. 1999; Weiss et al. 2006). For study populations exposed to relatively high levels of contaminants from fish consumption, namely, the Swedish Fishermen’s Families Cohorts and the New York State Angler Cohort, findings were also different. In Swedish fishermen’s sisters/wives who were highly exposed to PCBs from consumption of fatty fish from the Baltic Sea, no significant association was shown between PCBs in serum and time to pregnancy (TTP) (Axmon et al. 2000, 2001, 2002, 2004, 2006b). In the New York State Angler Cohort, increased TTP was found for women exposed to PCBs as measured by an index based on the amount of Lake Ontario fish consumed (Buck et al. 2000). Even in the New York State Angler Cohort, delayed TTP was not found when PCBs exposure was estimated by other methods (Buck et al. 1997, 1999; McGuinness et al. 2001). In addition, Law et al. (2005) found that TTP increased with increasing serum PCB levels in the Collaborative Perinatal Project, in which women were exposed to only background levels of PCBs. Therefore, whether PCB/PCDF exposure is associated with altered TTP in women remains uncertain.

In 1979, an unfortunate episode of mass poisonings occurred in central Taiwan after people used PCB-contaminated cooking oil; this incident involved about 2,000 people. Repeated heating of pyrolytic products of PCBs generated PCDFs, which also significantly contributed to the toxicity (Chen et al. 1985; Masuda et al. 1982). The illness was later called “Yu-cheng” (“oil disease” in Chinese). Severe health consequences have been documented from ingesting the compounds (Hsu et al. 1985), including chloracne, hyperkeratosis, gum hyperpigmentation, nail discoloration, Meibomian gland inflammation, and other symptoms similar to those reported for a poisoning accident that occurred in Japan in 1968 (Yusho episode). The Yucheng women were found to have more irregularity of menstruation, a higher prevalence of anemia, and increased stillbirth (Yu et al. 2000). Among girls born to Yucheng women, and therefore exposed *in utero* to PCBs/PCDFs, Yang et al. (2005) found irregular menstrual cycles, shorter mean duration of bleeding per cycle, and elevated serum levels of estradiol and follicle-stimulating hormone compared with an age- and neighborhood-matched reference group.

The purpose of our study was to determine whether fertility of Yucheng women (exposed to PCBs/PCDFs) was affected.

## Materials and Methods

### Study subjects

From 1979 to 1983, the Taiwan Provincial Department of Health registered 2,061 Yucheng victims (Hsu et al. 1985) using the following criteria: *a*) victims consumed brands of rice-bran oil produced in the factory known to be the source of the contamination; and *b*) skin, eye, and other symptoms developed during January–October 1979. About 10% of persons in the registry had a history of PCB exposure, were asymptomatic, and had elevated serum PCB concentrations (> 10 ppb, wet weight basis) by means of packed-column, electron-capture gas chromatography and an adaptation of the Webb–McCall method using Kanechlor 500 as reference standards (Guo et al. 1994). Serum samples from 56 women who were exposed in 1979 were collected in February 1992 and analyzed. In the serum samples, detectable levels of 2,3,4,7,8-pentaCDF and 1,2,3,4,7,8-hexaCDF were determined, with median values of 1,030 and 2,220 ng/kg serum lipid, respectively. The median of the total PCBs was 8,730 ng/kg on a whole weight basis, and the highest levels were found in PCBs 138, 153, 180, 170, and 156 (Guo et al. 1997). In addition, 20 years after the intoxication, 16 PCB congeners were analyzed by gas chromatography-electron capture detection in 435 Yucheng victims (414 adults and 21 children). The median concentration of total PCBs in adult serum was 1,500 ng/g lipid, with PCB-138 having the highest concentrations in Yucheng victims, still substantially higher than that of the general population in Taiwan (Lung et al. 2005).

It would have been ideal if, at the time of the poisoning, a community-based reference group could have been identified from the areas where the poisoning occurred and then followed in the same way as the exposed group. However, no such reference group was established at the time the accident occurred. We therefore used the 1979 addresses of the registry members as index addresses and attempted to identify the people who lived nearby in 1979 from the archives of the registration offices. We did this systematically beginning with the higher or lower numbered dwelling at random; we located the record of the current household, and, if that family had not been there in 1979, we worked through the archived records until we found the record of the family who had lived there. We then enumerated the family members and attempted to identify a control subject within the family. Reference subjects were required to be of the same sex and within 3 years of age of the index registry member, and they could not themselves be in the registry. If a household contained no eligible control subject, the next closest household was tried. In addition, we identified three referents for each registry member, and we attempted to contact the reference subject living nearest to the index address in 1979.

In 1993, we conducted a health survey of the Yucheng women and focused on menstrual characteristics and birth outcomes. The 1993 survey showed increased reproductive problems among Yucheng women, including a higher rate of abnormal menstrual bleeding and stillbirth (Yu et al. 2000). Thus, we conducted a follow-up study starting 1 July 2003 in which we invited women 25–45 years of age on that date (birth dates between 1 July 1958 and 30 June 1978) from the Yucheng cohort and their community reference group for reproductive history review by telephone interview. The interview, carried out using a structured questionnaire, was administered by telephone by trained interviewers who were unaware of the exposure status of the interviewed individuals. We obtained oral informed consent before the interview. Unmarried women were excluded from the analysis of TTP because in Taiwanese culture, unmarried women were not supposed to get pregnant. Those who had become pregnant before the time of exposure (before 31 December 1979) were also excluded. In addition, we planned to exclude women known to have problems with infertility noted before 1979, but no women reported having such problem.

### Data collection

The interview was carried out in 2003 by telephone to all eligible women to obtain information prior to their first pregnancy on factors potentially related to reproductive health. These questions included maternal and paternal ages at conception, maternal smoking and alcohol habits, body weight, height, body mass index (BMI), frequency of intercourse, and menstrual cycle length. Potential work exposure to reproductive hazards in the mother and father were also ascertained, including radiation, lead, organ solvents, and high temperatures.

### TTP

During the interview using the questionnaire, participating women were asked whether they were married. If the answer was “yes,” we collected the TTP (in months). The TTP was assessed for all pregnancies using a suite of questions: “Did you get pregnant the first month of trying?” “If not, how many months did it take you to get pregnant when you had normal sexual activity and no contraception?” The children’s birthday and gestational age were also collected. TTP was defined as the duration between the dates of discontinuing contraceptive measures and the beginning of the last menstrual period before pregnancy.

### Data analysis

Each woman was allowed to contribute only one pregnancy (the first pregnancy) to avoid interference from the correlation with the TTP of subsequent pregnancies. For analysis, if a woman became pregnant after marriage and before any menstruation, TTP was recorded as 1 month (Law et al. 2005). We used Kaplan–Meier survival curves to compare periods of not becoming pregnant between exposed women and controls, using the log-rank test. In the survival analysis, censoring of TTP was introduced following methods of Baird et al. (1986). Censoring was used for the following three conditions: *a*) TTP > 12 months; *b*) still having not become pregnant at the time of interview; and *c*) seeking medical assistance due to difficulty in getting pregnant before 12 months of trying. Among the eligible subjects, data for a total of 64 women were censored, including 58 women with TTP > 12 months, 2 women who were seeking medical treatment, and 4 women who still had not become pregnant at the time of interview.

Because TTP describes the time to an event, we used Cox regression, which has been suggested to be a suitable method for comparing populations with different exposures (Ecochard and Clayton 2000; Scheike and Jensen 1997). The Cox regression model translates into the Cox’s model for discrete time data, in which the likelihood equation is similar to that for the proportional odds model (Baird et al. 1986). Analyses of simulated TTP data have shown that the results estimated by logistic regression are similar to those estimated by Cox regression (Axmon and Hagmar 2005; Axmon et al. 2006a). The fecundability ratio (FR) describes the change in fertility when moving from one level of a factor to the next. For example, FR < 1 indicates a longer TTP compared with the reference group. In the present study, we used TTP as a dependent variable, and exposure group, maternal and paternal ages at conception, potential work exposure in mother and father, maternal smoking and alcohol habits, body weight, height, BMI, intercourse frequency, and menstrual cycle length as independent variables. Those variables with a *p*-value of ≥ 0.1 after the initial regression analysis were excluded, and only variables with a *p*-value < 0.1 remained in the final models. For dose–response analysis, we divided the Yucheng group into two levels by PCB serum concentrations in 1980–1982, categorized as low and high exposure.

Women who had noncontraceptive sexual activity for ≥ 12 months were included for logistic regression to determine odds ratios (ORs) for infertility (defined as TTP of > 12 months). Potential confounders listed for Cox regression (see above) were included in the regression analysis. These variables were erased one at a time by a backward stepwise procedure, and those variables with a *p*-value of < 0.1 remained in the regression analysis. No couple received fertilization treatment before attempting pregnancy for at least 12 months.

## Results

**Figure 1:**
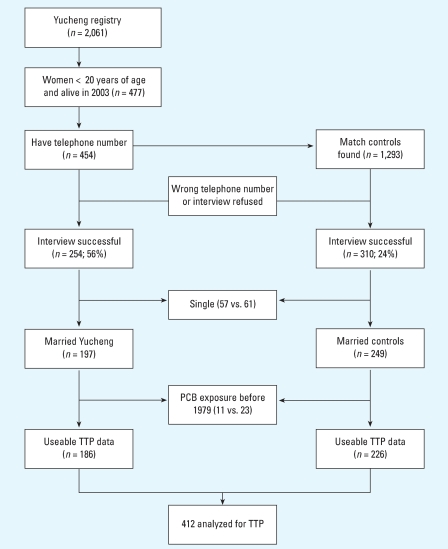
Recruitment and participation of the study populations in central Taiwan, 2003.

A total of 564 subjects completed the telephone interview satisfactorily. The participation rate was higher for the Yucheng women than for the reference group (56% vs. 24%). However, we did not find a significant difference in the age distribution between those participating and those not participating in the Yucheng group (39.7 ± 4.4 and 39.3 ± 6.2 years, respectively) and the reference group (39.1 ± 4.7 and 39.5 ± 5.9 years, respectively). Among those participating in the telephone interview, 446 were married and provided a reproductive history. Eleven Yucheng women and 23 controls had been pregnant before 31 December 1979, and were thus excluded from the analysis, leaving 412 women in the TTP study. Figure 1 depicts the recruitment and participation of Yucheng and reference women in this study. Table 1 summarizes the demographic characteristics of the 412 Yucheng and reference women. Yucheng women had lower height and weight than reference women. A greater percentage of Yucheng women ever used alcohol. The frequency of intercourse before the first pregnancy was lower among Yucheng women than among reference women.

**Figure 2:**
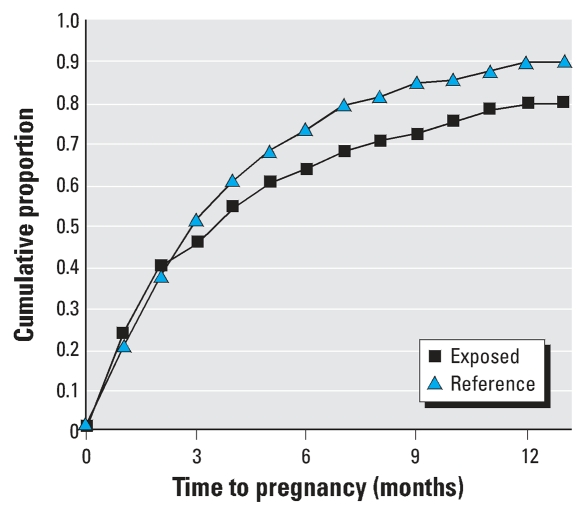
Cumulative distribution of TTP in Yucheng women (*n* = 149; median = 4 months) and reference women (*n* = 203; median = 3 months). *p*-Value of log-rank test = 0.019.

Figure 2 shows the cumulative distribution of TTP for both the Yucheng and reference groups. TTP was longer in Yucheng women (*p* = 0.019), indicating that Yucheng women had more difficulty in becoming pregnant. The median TTP in Yucheng women was 4 months, compared with 3 months in reference women.

The results of the Cox regression are shown in Table 2. The adjusted FR was 0.90 [95% confidence interval (CI), 0.80–1.00] for Yucheng women compared with the reference group. When Yucheng women were divided into high- and low-exposure groups according to 1980–1982 serum PCB levels, FRs were not significantly different from those of the reference group.

Logistic regression results are shown in Table 3. Among reference women, 9.7% failed to conceive after 12 months, whereas 19.7% of Yucheng women failed to conceive. The crude OR of infertility was 2.26 (95% CI, 1.29–4.05) and the adjusted OR was 2.34 (95% CI, 1.23–4.59) for Yucheng women compared with reference group. This was essentially unchanged after adjusting for potential confounders (i.e., paternal and maternal age, frequency of intercourse). In this analysis, paternal age > 30 years was also a risk factor for infertility (adjusted OR = 3.25; 95% CI, 1.25–8.44).

We examined the relationship between the frequency of intercourse and infertility in the logistic regression analysis and found no statistical significance.

## Discussion

The present study is the first study on fecundability in women highly exposed to PCBs and PCDFs. We studied time to the first pregnancy among women exposed to PCBs and PCDFs and compared them with unexposed, age-matched neighbors. We found a prolonged TTP for Yucheng women and an increased risk of infertility, as defined by no pregnancy after 12 months of normal sexual activity using no contraception.

Our findings were consistent with previous studies of the New York State Angler Cohort (Buck et al. 2000) and the Collaborative Perinatal Project (Law et al. 2005). Buck et al. (2000) found that maternal consumption of fish for 3–6 years was associated with reduced fecundability (FR = 0.75; 95% CI, 0.59–0.91), as was consuming more than one fish meal per month in 1991 (FR = 0.73; 95% CI, 0.54–0.98). In the Collaborative Perinatal Project, Law et al. (2005) found an association between TTP and serum PCB levels in the third trimester of pregnancy.

Different results on TTP have been reported among people exposed to PCBs and related chemicals. In the Swedish fishermen’s families cohort (Axmon et al. 2000, 2001, 2002, 2004), slightly shortened TTP was associated with high PCB-153 concentrations in serum from the fishermen’s wives and sisters (FR = 1.75; 95% CI, 1.05–2.09). In addition, among women from Greenland, there seemed to be an association between PCB exposure and prolonged TTP (FR = 0.73; 95% CI, 0.54–0.97) (Axmon et al. 2006b). In studies in which fish intake was the main source of PCB exposure, potential beneficial effects of fish intake might have counterbalanced the effects of PCBs. However, in the Yucheng cohort in the present study, such beneficial effect of fish intake was likely not different from that of the reference group. We therefore believe that the detrimental effects of PCB/PCDF exposure on fecundibility was real.

Other environmental chemicals could potentially affect fecundability. Cole et al. (2006) examined the effects of mercury, benzene hexachloride, dichlorodiphenyltrichloro-ethane/dichlorodiphenyldichloroethylene (DDT/DDE), hexachlorobenzene, oxy-chlordane, and PCBs on TTP, and found that only mercury was associated with prolongation of TTP. In the 41 couples they studied, PCB exposure levels were much lower than in the Yucheng individuals. Therefore, the findings of Cole et al. (2006) did not preclude possibilities of reduced fecundability by PCBs/PCDFs, as has been found in our study.

There are many potential mechanisms by which environmental agents can affect fecundability and pregnancy outcomes, as suggested by animal or *in vitro* studies (Silbergeld and Patrick 2005). Exposure before conception might affect pregnancy by hormonal dysregulation or oocyte toxicity (Silbergeld and Patrick 2005). Measurable concentrations of PCBs are found in the follicular fluid of women (Trapp et al. 1984). Krogenaes et al. (1998) reported that exposure to both noncoplanar PCB-153 and coplanar PCB-126 during *in vitro* maturation had adverse effects on bovine oocytes, reducing the percentage of oocytes able to complete the first mitosis after fertilization, and decreasing the ability of oocytes to mature and sustain subsequent embryo development. After fertility, pregnancy may fail because of the loss of the embryo or fetus. Thus, subfertility can also be caused by undetected early pregnancy loss (Chard 1991; Wilcox et al. 1999). PCBs, and PCDFs can bind to the AhR and induce estrogenic and antiestrogenic effects mediated by AhR-dependent mechisms (Hombach-Klonisch et al. 2005). Dioxin-like chemicals have been reported to reduce both progesterone and estradiol secretion in porcine granulosa cells by altering the activity of enzymes involved in steroid biosynthesis (Gregoraszczuk 2002). Decreased progesterone may reduce the implantation rate and alter the maintenance of corpus luteum. Rodents exposed to PCBs experience a reduced number of implantation sites, embryo toxicity, and reduced litter size (d’Argy et al. 1987; Linzey 1988; Ronnback and de Rooij 1994). PCB/PCDF-induced subfertility might be caused by interfered ovarian function, ovulation, corpus luteum maintenance, implantation, and embryo development.

The present study has several strengths. First, our study was a cohort study with long-time follow-up; thus, we were able to assess the chronic effect on female fertility after exposure to persistent organochlorine compounds. Second, the high exposure to PCBs and PCDFs allows for examination of the effects of these compounds. Third, we used an unexposed reference group matched by age, sex, and area of residence. Fourth, while interviewing the subjects, the interviewers were unaware of the exposure status. Therefore, we were able to examine health effects by comparing the two groups with similar background factors; the effects of PCB/PCDF exposure seemed to be the single most important factor affecting health effects between the two groups.

The present study also has limitations. For example, there was a relatively low participation rate of the Yucheng and reference groups partly because of a long follow-up time of 24 years. Also, there was limited willingness to participate in a study on reproductive issues, which are considered rather personal in Taiwan. The sample size was borderline for providing sufficient statistical power, thus hindering further stratified analysis. Another weakness of our study is the retrospective recall of TTP. The long period might have introduced difficulties in recall. However, such difficulties were not different between the exposed and reference groups, because the average recall times were similar for both groups (approximately 14 ± 6 years). Therefore, no differential error was introduced because of difficulty in recall.

Nguyen and Baird (2005) previously showed that men tended to underestimate TTP (mean difference of –1.2 cycles) compared with spouses. However, among men who had fathered only one child, such underestimation was not severe (Nguyen and Baird 2005). In the present study, we asked women about TTP only for the first pregnancy; therefore, the underestimation should have been minimal. We did not ask whether the pregnancy was planned. However, we specifically asked “For how long have you tried to conceive before you became pregnant?” Those who did not attempt to conceive would have answered “I have never tried to conceive.” However, among our study participants, no one told us that she did not attempt to conceive.

It would have been biologically plausible and worth investigating to assess the association between PCBs and TTP stratified by age at first PCB exposure; however, with a limited number of subjects, this would lead to small sample size in each stratification, thus not allowing for adequate power for testing the hypothesis of this study. Equally, there was a limited number of subjects for which we could examine fertility effects stratified by time of exposure, such as before or after puberty.

Although some of the Yucheng women were exposed before menarche, the persistent nature of the contaminants to which they were exposed caused chronic internal exposure. It is uncertain whether resulting effects were different based on exposure during childhood or adolescence compared with exposure during adulthood. However, because of the relatively small number of women exposed, we did not have adequate power for stratified analysis looking at differential effects of exposure before and after menarche.

TTP is a direct and valid measure of human fecundity by both questionnaire and interview. Previous authors have addressed the validity and reliability of a questionnaire for TTP and the use of the Cox regression model (Baird et al. 1991; Joffe 1997; Joffe et al. 1993). The applicability of using TTP to study environmental exposure has also been demonstrated (Baird et al. 1986). TTP studies need to take several population characteristics into consideration, especially if the compared populations differ in factors such as desired family size, use of contraceptive methods, and sexual activity (Bonde et al. 2006). Frequency of sexual activity was included in our regression models, and thus was adjusted. We used the first pregnancy of married women for TTP comparison to control for the effect of desired family size. When women were asked if they desired no children, none of our subjects gave a positive answer. Also, < 15% of Yucheng and reference women ever used contraceptive methods. Because of the multiplicity of methods, we did not include contraception in the final analysis. Nevertheless, such effects might have been minimal because of the similar and small proportion of contraception use in the two groups.

The reported TTP seems to vary greatly in different countries. For example, the proportion of women in our reference group who were not able to conceive after 12 months was lower than that reported for European populations (9.3% vs. 16%) (Juul et al. 1999). However, the median TTP in the reference group of the present study was similar to the reference group in another Taiwanese population (Shiau et al. 2004). Cultural differences in trying to conceive and in reporting TTP might have contributed to this phenomenon (Bonde et al. 2006).

It is possible that Yucheng women were less willing to have children, which might have caused longer TTP in the present study. However, data show that this might not be the case, or if it is, the effect is not significant. When asked “have you ever decided to avoid pregnancy due to physical conditions?” only one Yucheng women and none of control women answered “yes.” However, the Yucheng woman questioned had three children at the time of the interview. She married at 28 years of age, had a TTP of 4 months, and had the first child at 29 years of age. In addition, the mean age of first childbirth was not different between Yucheng women and the reference group.

Previous diethylstilbestrol (DES) exposure in men and women is known to affect reproductive systems and reproductive outcomes. However, the use of DES in gestation was not a common practice in Taiwan. Therefore, we did not ask women or spouses in the present study about such exposure.

We chose to analyze the TTP of the first pregnancy instead of the TTP of all pregnancies. A women’s body burden of PCBs/PCDFs is reduced when she is breast-feeding (Ryan et al. 1994), so the first pregnancy is the most exposed. To avoid interference from previous pregnancies, we used only the first TTP for analysis.

In conclusion, we found prolonged TTP and reduced fertility among women previously exposed to PCBs and PCDFs. Because of the limited sample size and the 10% drop in fertility rate, the results need be interpreted with caution. Further investigation with longer follow-up and a larger population are warranted.

## Figures and Tables

**Table 1:** Background characteristics of Yucheng (*n* = 186) and reference (*n* = 226) women, 2003.

Characteristic^a^	Yucheng	Referent	*p*-Value
Age in 2003 [years (mean ± SD)]	39.6 ± 4.5	39.2 ± 4.7	0.51
Height [cm (mean ± SD)]	156.7 ± 5.1	158.0 ± 5.1	0.014
Body weight [kg (mean ± SD)]	55.9 ± 8.1	57.6 ± 9.0	0.06
BMI (mean ± SD)	22.8 ± 3.4	23.1 ± 3.2	0.41
Education [no. (%)]
< High school	97 (53.0)	105 (47.5)	0.44
High school	63 (34.4)	80 (36.2)	
> University	23 (12.6)	36 (16.3)	
Ever smoke [no. (%)]
Yes	5 (2.7)	1 (0.5)	0.10
No	178 (97.3)	220 (99.6)	
Ever drink alcohol [no. (%)]^b^
Yes	7 (3.8)	1 (0.4)	0.03
No	176 (96.2)	219 (99.6)	
Women’s age at first pregnancy [years (mean ± SD)]^c^	25.2 ± 3.4	24.9 ± 3.5	0.42
Husband’s age at first pregnancy [years (mean ± SD)]^c^	27.8 ± 3.8	27.5 ± 3.9	0.33
Intercourse before first pregnancy [times/week (mean ± SD)]	2.1 ± 1.1	3.0 ± 2.2	< 0.0001
Age at menarche [years (mean ± SD)]	13.9 ± 1.4	13.9 ± 1.4	0.76
MC before first pregnancy [days (mean ± SD)]	29.5 ± 7.2	29.9 ± 6.8	0.58
Women employed before first pregnancy [no. (%)]
No	32 (17.3)	37 (16.5)	0.83
Yes	153 (82.7)	187 (83.5)	
Risk of exposure from husband’s occupation [no. (%)]			
No	157 (88.2)	197 (88.3)	0.97
Yes	21 (11.8)	26 (11.7)	
Used contraceptive methods [no. (%)]^d^
Never	155 (85.6)	188 (85.4)	0.96
Ever	26 (14.4)	32 (14.6)	

MC, menstrual cycle.

a Unpaired student *t*-test or chi-square test.

b Fisher’s exact test.

c Only a subgroup (Yucheng = 179; referents = 217) of study participants ever became pregnant.

d Only a subgroup (*n* = 401) of study participants ever used contraceptive methods.

**Table 2:** FRs (95% CIs) between reference and Yucheng women and among 1980–1982 serum PCB groups.

Subject group	No.	Censored	Crude FR (95% CI)	Adjusted^a^ FR (95% CI)
All subjects (*n* = 412)				
Reference	226	24	1	1
Yucheng	186	40	0.88 (0.79–0.98)	0.90 (0.80–1.00)
With serum PCB concentration (*n* = 369)				
Reference	226	24	1	1
Low PCB exposure (< 46 ppb)	71	14	0.79 (0.58–1.05)	0.80 (0.59–1.10)
High PCB exposure (≥ 46 ppb)	72	14	0.85 (0.62–1.13)	0.86 (0.63–1.16)

a Adjusted for intercourse frequency, women’s age at first pregnancy, and husband’s age at first pregnancy.

**Table 3:** Crude and adjusted ORs for infertility due to exposure group, age of woman and husband at first pregnancy, and frequency of intercourse before first pregnancy in 408^a^ women.

Variable	No.	Infertility^b^ [*n* (%)]	Crude OR (95% CI)	Adjusted OR^c^ (95% CI)
PCB exposure				
Reference	225	22 (9.7)	1	1
Yucheng	183	36 (19.7)	2.26 (1.29–4.05)	2.34 (1.23–4.59)
Women’s age at first pregnancy (years)				
< 25	190	15 (7.9)	1	1
25–28	121	14 (11.6)	1.53 (0.70–3.30)	1.13 (0.47–2.66)
> 28	85	24 (28.2)	4.59 (2.28–9.50)	1.81 (0.68–4.98)
Husband’s age at first pregnancy (years)				
< 28	216	14 (6.5)	1	1
28–30	69	11 (16.0)	2.74 (1.16–6.34)	2.24 (0.85–5.74)
> 30	110	28 (25.4)	4.93 (2.51–10.08)	3.25 (1.25–8.44)
Frequency of intercourse before first pregnancy (times/week)				
2–5	246	29 (11.8)	1	1
< 2	116	17 (14.7)	1.28 (0.66–2.42)	1.28 (0.64–2.52)
> 5	31	5 (16.1)	1.44 (0.46–3.77)	2.46 (0.72–7.33)

a Four couples who left the study early were excluded.

b Couples who tried to conceive for 12 months without success.

c Adjusted for PCB exposure, intercourse frequency, women’s age at first pregnancy, and husband’s age at first pregnancy.
